# Wearable energy systems: what are the limits and limitations?

**DOI:** 10.1093/nsr/nwac060

**Published:** 2022-03-31

**Authors:** Lu Yin, Joseph Wang

**Affiliations:** Department of Nanoengineering, Center for Wearable Sensors, University of California San Diego, USA; Department of Nanoengineering, Center for Wearable Sensors, University of California San Diego, USA

## Abstract

With over a decade of tremendous effort and exciting developments, we have yet to see the successful commercialization of wearable soft electronics due to the lack of reliable energy solutions. This perspective summarizes the theoretical limits and the practical limitations for wearable energy devices and proposes strategies to address such limitations.

The recent decade marks a sharp rise in wearable electronics with a market size approaching 100 billion USD. Alongside this boom in consumer wearable electronics, researchers have pursued, during the past 15 years, next-generation wearable electronics featuring thin, soft, conformal, flexible and stretchable form factors for application in advanced human–machine interaction and comprehensive sensing. These efforts have conceived a wide class of wearable sensors, displays, integrated circuits, batteries, supercapacitors and energy harvesters, that assemble into an ecosystem of future wearable devices [1,2]. Similar to other types of electronics, wearable electronics require a constant power source for uninterrupted operation. As the demand for wearable electronics grows, so does the demand for wearable power sources. Excitingly, the concept of self-powered wearable systems has recently gained increasing popularity, entailing the integration of energy harvesters and energy storages devices with wearable electronics, towards energy-autonomous extended on-body operation [1]. While research into wearable energy-harvesting and storage devices, and the integration thereof, is soaring, to this day researchers have not been able to meet the energy demands of the majority of existing wearable applications. As examples, wireless earbuds or smartwatches use 300–1500 mWh batteries, while most flexible batteries feature <5 mWh/cm^2^ energy; low-power microcontrollers for wearable sensors typically require 1–100 mW power input, while wearable bioenergy harvesters can only harvest <1 mW/cm^2^ power [1]. Such major gaps between energy demand and supply become the bottleneck of the ecosystem. To date, few wearable electronics possessing ubiquitous conformity and flexibility, and that are compatible with diverse lifestyles, exist on the market. While the performance of wearable energy devices has certainly not reached its full potential, many practical factors hinder the development of next-generation commercially viable wearable electronics. This perspective aims to stimulate discussion on the theoretical limits and practical limitations of wearable energy devices, with a view to addressing these major issues (Fig. 1).

Many electrochemical energy storage devices, predominantly batteries, and supercapacitors have been developed into soft wearable form factors [3]. While some compromises between mechanical flexibility and electrical performance must be made, wearable energy storage devices with high power and energy density have been reported, including Li-ion batteries (538yWh/L), Zn batteries (300yWh/L) and supercapacitors (88.1 Wh/L), which are close, in terms of performance level, to their commercial, non-wearable rigid counterparts [4–6]. In addition, battery and supercapacitor technologies still have plenty of room for improvement with regard to approaching the theoretical limit of the specific energies for Li-air metal batteries and Zn-air batteries (ca. 5 Wh/g and 1 Wh/g, respectively), which may also be obtainable one day for wearable batteries [7]. On the other hand, a myriad of energy-harvesting devices—based on photovoltaic, electromagnetic, piezoelectric, triboelectric, thermoelectric and electrobiocatalytic energy-conversion mechanisms—have also been developed [8]. The integration of multiple harvesting mechanisms into a ‘wearable microgrid’ can potentially maximize the energy that is harvestable using wearable devices, with the theoretical limit of harvesting a few hundred watts from bodily movements as well as from sunlight and heat [1]. This would allow, potentially, hundreds of watt-hours of energy to be harvestable from wearables, which could sustain the continuous and autonomous operation of the majority of consumer electronics on the market. Even if only 50% of the theoretical energy density of batteries and only 1% of the theoretical efficiency for energy harvesters were reached, their performance would still be practical and attractive for commercialization. However, the reality is that many practical limitations still exist, and we are still very far from reaching these fundamental limits of wearable energy systems.

To our knowledge, the most immediate limitation of soft wearable electronics is the lack of a high-performance flexible battery. While many companies have developed small, safe, thin and flexible batteries, they are characterized by low energy (below 100 mWh) and low power (below 10 mW), due to trade-offs between compactness and flexibility of structure (e.g. kirigami/origami), or between the electrochemical and mechanical performance of materials.

**Figure 1.**
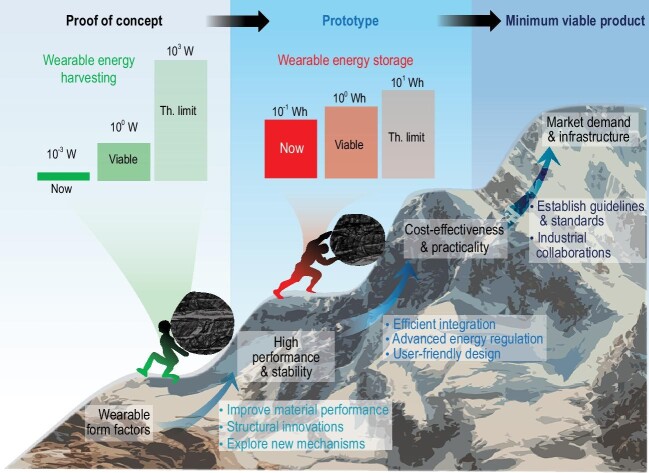
Theoretical limits of performance, practical limitations and potential solutions for next-generation wearable energy-harvesting and storage devices, from proof of concept to commercialization.

These small flexible batteries are thus unable to power existing integrated circuits (microcontrollers, microprocessors, system-on-chip, etc.) with basic computing, storage and wireless transmission capabilities that typically require 20–100 mW power and an active runtime of several hours at least [1]. Although flexible batteries with performances comparable to that of existing Li-ion or Li-metal coin cells have been reported recently [3,9], their commercialization may still face many practical limitations. As of now, most wearable original electronics manufacturers (OEMs) elect to use bulky and non-flexible Li-ion or lithium-polymer pouch cells due to their stable performance, abundant supply and well-established ‘infrastructures’, including their form factors, connection conventions and energy management solutions. Soft battery technologies usually do not deliver sufficient performance and lack the established infrastructure. As a result, current OEMs have little incentive to adopt new flexible energy-storage technologies or new technologies that face very low immediate market demand and have high research and development (R&D) costs with regard to developing the auxiliary ecosystem, which forms a technological gridlock.

After over a decade of development, wearable energy harvesters are still limited significantly in their performance and practicality. Despite frequent media coverage about various attractive wearable prototypes, few wearable products with integrated energy harvesters are commercially available for practical consumer use. While countless wearable energy harvesters with great mechanical flexibility, stretchability and conformity have been reported, their areal powers are below 5 mW per cm^2^ and their total harvestable energy is only <10 mWh per day, making them impractical for most low-power wearable applications. The conversion of these thermal, biomechanical and biochemical energies into electrical energy is characterized by extremely low efficiency, due to limitations in both the bulk material performance, device architecture and their respective energy regulation. The use of these energy sources in most wearable systems is characterized by intermittent supply, and demands constant high-frequency movement, high temperature gradient or heavy sweating, therefore they are deemed to be low ‘energy return-on-investment’, non-user-friendly and impractical [10]. Wearable energy harvesters that work continuously and lead to high levels of electrical energy generation are required. Beyond these, many harvesting devices have limitations in cost, scalability, biocompatibility, washability and long-term durability, which restrict their practical viability in commercial wearable systems.

The development of next-generation wearable electronics hinges heavily on addressing the challenges involved in approaching the theoretical limits of wearable energy systems. Encouragingly, the current trend for developing product-like integrated wearable systems has promoted interdisciplinary collective efforts in material and structural engineering, electrochemistry, power regulation, energy harvesting and storage, circuit design and programming, product design, and manufacturing. Furthermore, recent developments in ultra-low-power electronics, long-range wireless power delivery and dual-function devices (e.g. photo-rechargeable batteries, self-charging biosupercapacitors and self-powered sensors) can revolutionize the current design concepts of integrated wearable systems. We believe that such multidisciplinary efforts will identify and address the missing links in the wearable ecosystem. In addition, extensive efforts should be directed towards improving device performance, efficiency and durability, as well as exploring new energy-harvesting and storage mechanisms in order to achieve significantly higher energy outputs. Universal testing, design guidelines, standards and reporting protocols on flexibility, stretchability and washability (similar to those of certifying solar cells), should be established for fair assessment and proper comparison of the performance of wearable energy-harvesting and storage devices. Upper-level efforts channeling industrial R&D with academic research output can facilitate the breaking of the technological gridlock, ensuring the market-demand-guided development of wearable energy devices, and aiding OEMs’ transition from legacy energy solutions to the next generation of efficient and practical wearable energy systems. With these efforts, we trust that next-generation wearable soft electronics will morph from the hype of today to the practical reality of tomorrow.
